# Qualitative research in cardiac surgery: importance, challenges, and opportunities

**DOI:** 10.1007/s12055-025-01923-w

**Published:** 2025-02-25

**Authors:** Eka Narayan, Ramsah Cheah

**Affiliations:** Hull York Medical School, Allam Medical Building, University of Hull, Hull, HU6 7RX UK

**Keywords:** Qualitative research, Cardiac surgery, Quantitative research

## Abstract

Qualitative research in cardiac surgery remains underutilised despite its crucial role in understanding patient experiences and improving care delivery. Cardiac surgery has traditionally been dominated by quantitative research and despite its clear value, qualitative research remains underutilised in cardiac surgery. This narrative review highlights key qualitative findings in valve replacement procedures, including patient autonomy in decision-making, post-operative challenges, and financial burdens. Mixed-methods research combining quantitative and qualitative approaches offers a comprehensive framework for addressing complex healthcare challenges. We propose strategic areas for future qualitative research and recommend increased integration of qualitative methodologies in cardiac surgical practice.

## Introduction

Cardiac surgery has traditionally been dominated by quantitative research, focusing on measurable outcomes such as mortality rates, morbidity, operative times, and complication rates. While these metrics are fundamental for evaluating surgical techniques and patient outcomes, they tell only part of the story. The field’s heavy reliance on quantitative measures has often overlooked the crucial subjective experiences of patients, families, and healthcare providers which are captured by qualitative studies. The number of qualitative studies in cardiac surgery published in PubMed-indexed journals over the last decade was 978 (0.49%) out of a total of 196,210 qualitative studies across all fields. In comparison, qualitative research in other specialties such as orthopaedics (*n* = 3335) and neurology (*n* = 3228) was over three times higher, highlighting the relative underrepresentation of qualitative research in cardiac surgery.

Qualitative research is an approach that explores phenomena through non-numerical data to understand experiences, behaviours, and processes. It is particularly useful in exploring complex or poorly understood issues, generating hypotheses, and providing context for quantitative findings [1]. In addition, the mixed-model approach for conducting research is also very useful for research. The mixed-model approach integrates both numerical and descriptive approaches to provide statistical results with contextual analysis [1]. A detailed differentiation between quantitative, qualitative, and mixed-model research methodology has been provided in Table 1.

**Table 1:** Comparison of quantitative, qualitative, and mixed methods

Aspect	Quantitative methods	Qualitative methods	Mixed methods
Definition	Focuses on measuring variables and testing hypotheses using numerical data	Aims to explore and interpret experiences, behaviours, and social contexts through descriptive data	Integrates both numerical and descriptive approaches to provide a fuller understanding
Purpose	To quantify relationships, test predictions, and derive generalisable conclusions To find out “how much,” “how often,” or “what causes what” using data	To gain deeper insights into complex or poorly understood issues To explore “why” or “how” something happens in depth	To merge statistical results with contextual analysis for richer insights To answer both “how much” and “why” questions together for a complete understanding
Data collection	Structured tools like surveys, clinical databases, experiments	Unstructured or semi-structured approaches like interviews, notes, and textual analysis	Uses a combination of structured (e.g. surveys) and unstructured (e.g. interviews) methods
Data type	Numeric data, such as percentages, means, or statistical outputs	Descriptive data, including narratives, observations, or multimedia content	Both statistical figures and descriptive accounts
Analysis	Relies on statistical techniques like regression, correlations, and hypothesis testing	Employs coding techniques, pattern recognition, and thematic analysis to draw meaning	Combines statistical computations with thematic or narrative synthesis
Strengths	Effective for identifying trends and testing causation. Results are easily standardised and compared	Captures nuances and depth in human behaviour or processes	Provides both numerical precision and contextual depth. Triangulates findings for comprehensive understanding
Limitations	May oversimplify complex phenomena. Dependent on large sample sizes and data quality	Limited in generalisability due to small, context-specific samples Can be subjective if not carefully managed	Requires expertise in both methodologies. Resource-intensive and time-consuming to conduct and analyse
Examples in cardiac surgery	Assessing risk factors for outcomes. Comparing hospital performance using observed-to-expected mortality ratios	Exploring patient decision-making for high-risk surgeries. Examining teamwork and communication in surgical settings	Combining patient interviews with quantitative data on surgical outcomes

## Importance of research

Incentivising publishing through tangible benefits like research grants, awards, and promotion-based metrics can encourage greater participation, particularly in academic institutions, while corporate hospitals can foster a publication culture through funding, recognition, or bonuses. Publishing not only promotes self-audit, enabling surgeons to evaluate and improve techniques, but also fosters professional growth and innovation. Institutional policies, such as requiring publications for memberships of professional bodies, and structured work environments with dedicated academic days, can inspire productivity and balance research with clinical duties. Publishing is crucial for professional relevance, advancing medical knowledge, and improving patient care, which institutions can support through mentorship programs, collaborative research, and co-authoring initiatives. Practical strategies include allocating dedicated research time, providing administrative and statistical support, and conducting writing workshops, ensuring a balanced approach to academic and clinical responsibilities. Addressing challenges, such as the pressure of balancing publishing with practice, requires collaboration with academic institutions, shared research resources, and incentivised group publications to distribute workloads and enhance participation.

## Importance of qualitative research

Qualitative research fills this critical gap by providing insights into the human aspects of cardiac care that numbers alone cannot capture. This includes understanding patient experiences throughout their surgical journey, from preoperative anxiety to post-operative adaptation, as well as the perspectives of caregivers and healthcare providers. For instance, a qualitative study in children with hypoplastic left heart syndrome revealed nuanced aspects of patient care that quantitative analysis alone could not identify, though quantitative methods proved more useful for prognostication [2]. The integration of both research approaches is essential for comprehensive patient care. While quantitative measures like survival rates after coronary artery bypass grafting (CABG) indicate procedural success, they fail to capture crucial aspects such as quality of life, satisfaction with care, and emotional well-being. Qualitative research provides the context and depth needed to develop more effective, patient-centred interventions.

## Conduct of qualitative review

Qualitative research relies on methods like interviews, focus groups, observations, and document reviews to gather detailed insights into experiences, behaviours, and contexts. Data is collected systematically, often using recordings and transcripts to ensure accuracy and comprehensiveness. Iterative analysis begins during data collection, where information is reviewed, categorised, and coded to identify patterns and themes. Analytical tools help manage large datasets, with the ultimate goal of generating frameworks or theories that explain underlying relationships and processes.

Reporting in qualitative research emphasises clarity and transparency, with detailed descriptions of study design, sampling, data collection, and analysis. Findings are often supported by direct quotes from participants to provide depth and authenticity, helping to place the results in a meaningful context. To ensure rigor, researchers validate their findings through triangulation, participant feedback, and maintaining detailed documentation of all steps in the process. Providing rich descriptions of the study context enhances transferability, ensuring the research can inform broader applications or comparisons. This will include practical examples of qualitative data collection methods (e.g. interviews, focus groups), analytical strategies (e.g. thematic analysis), and ensuring trustworthiness and rigor (e.g. member checking, triangulation).

## Challenges in qualitative studies in cardiac surgery

Despite its clear value, qualitative research remains underutilised in cardiac surgery. To gain a perspective on the underutilisation of qualitative research, it was found that over a 20-year period, only 15 journals out of 33 cardiovascular journals published qualitative studies. During the same study period, only 365 qualitative studies were published in these journals with 78% being published in 3 nursing journals [3]. This gap stems from several factors: the historical dominance of the biomedical model, which prioritises objective measurements; a perception that qualitative research is less rigorous despite its established methodologies; and limited expertise in qualitative methods among cardiac surgeons and researchers.

## Important qualitative studies in cardiac surgery

Some of the important qualitative studies carried out in cardiac surgery centre around Heart valve replacement procedures. The quantitative randomised controlled trials among low, intermediate, and high risk assess the non-inferiority of transcatheter aortic valve replacement (TAVR) in comparison with surgical aortic valve replacement (SAVR) [4]. However, it is the qualitative studies that are required to evaluate patient autonomy and decision-making, financial and logistical burdens, patient experiences, and quality of life in these patients.

In a qualitative study exploring the conditions for autonomous decision-making among older adults undergoing TAVR, the study revealed that while patients often experienced ambivalence and dependency on physicians’ advice, they also expressed a sense of self-empowerment in making their decisions. The study highlighted the importance of shared decision-making tailored to older adults’ unique needs [5]. This was further illustrated in another recent study that investigated patients’ willingness to participate in decision-making regarding anticoagulation therapy post-heart valve replacement [6].

Qualitative studies have also evaluated a telephone-based intervention that has been shown to effectively reduce patient anxiety [7]. Persistent sleep disturbances and vivid memories of delirium episodes were reported even 4 years post-treatment and qualitative studies have shown that octogenarian patients’ emotional distress during hospitalisation has been a recurring theme, necessitating greater attention to sleep management and psychological support for older patients [8].

An important study in India examined the out-of-pocket expenditure (OOPE) and challenges faced by patients during follow-up care in South India. The study found that over 60% of patients incurred catastrophic health expenditures, with significant financial burdens exacerbated by travel and the need for accompanying caregivers. The authors recommended the adoption of telemedicine to decentralise follow-up care, potentially alleviating these challenges [9]. Once the patient is discharged from the hospital, quantitative studies focus mainly on re-admission rates, late mortality, and re-intervention. Qualitative studies are needed to understand the lived experiences of patient’s post-heart valve replacement and have been shown to capture a “difficult life” characterised by ongoing physical, emotional, and social challenges. It has been shown that patients struggle with lifestyle adjustments after cardiac surgery, highlighting the need for comprehensive post-operative care programs addressing both physical recovery and emotional well-being [10].

## Opportunities for qualitative research in cardiac surgery

A non-exhaustive summary of some of the opportunities that are available in cardiac surgery has been enumerated in Table 2. Researchers can benefit from collaborating with the Patient-Centered Outcomes Research Institute (PCORI) which plays a pivotal role in funding and advancing quality-oriented research, particularly in healthcare settings. Established to enhance evidence-based decision-making, PCORI funds studies that prioritise patient-centred outcomes, ensuring that research addresses questions relevant to patients, caregivers, and clinicians. Its focus on comparative effectiveness research empowers healthcare professionals to evaluate the benefits and risks of various interventions, fostering improved clinical practices and patient care [11].

**Table 2:** Examples of qualitative research ideas in cardiac surgery

Area of focus	Qualitative research idea	Methodology
Patient decision-making	Explore factors influencing patients’ choices for high-risk surgeries	Conduct semi-structured interviews with patients pre- and post-surgery to understand their concerns and priorities
Team communication in the OR	Study how communication dynamics among surgical team members affect patient outcomes and error rates	Use direct observation and audio or video recordings of surgical team interactions during procedures
Impact of surgical complications	Investigate how patients and families cope with major surgical complications or adverse events	Conduct focus groups with families and interviews with patients experiencing complications
Barriers to early surgical intervention	Examine why patients delay seeking surgical treatment for conditions like endocarditis or coronary disease	Use patient narratives collected through interviews to identify recurring themes
Post-operative recovery experiences	Understand the recovery challenges faced by cardiac surgery patients, particularly in the elderly population	Conduct longitudinal interviews with patients during the first 6 months post-surgery
Technology adoption	Assess surgeons’ perspectives on integrating robotic or minimally invasive techniques in practice	Organise focus groups with surgeons to explore perceptions of new technology, training, and barriers
Patient education and consent	Study the effectiveness of current consent and education processes for high-risk cardiac procedures	Interview patients and caregivers about their understanding of risks and benefits pre-surgery
Burnout among cardiac surgeons	Explore factors contributing to burnout and its impact on professional performance and decision-making	Conduct interviews with surgeons to identify stressors and coping mechanisms
Ethical decision-making	Investigate how surgeons handle ethical dilemmas in resource-limited settings	Use case studies and interviews to analyse decision-making processes
Health disparities	Study disparities in access to cardiac surgery based on socioeconomic or geographic factors	Interview underserved patients and compare with those in urban centres to highlight systemic challenges

By offering substantial funding and emphasising methodological rigor, PCORI encourages high-quality, outcome-driven studies that address real-world challenges. Its support for initiatives like shared decision-making tools and patient engagement frameworks aligns closely with the goals of quality-oriented research, enabling professionals to publish findings that are both impactful and clinically relevant. Integrating PCORI-backed approaches into institutional frameworks can further inspire professionals to engage in meaningful, patient-focused research.

## Conclusion

Qualitative research is an essential complement to quantitative studies in cardiac surgery. By focusing on the human experiences and contextual factors that shape care delivery, it can address gaps in knowledge and contribute to more holistic, patient-centred approaches. To advance this agenda, cardiac surgery researchers must embrace interdisciplinary collaborations, invest in training for qualitative methods, and prioritise studies that address the lived experiences of patients and providers. By doing so, the field can achieve a more comprehensive understanding of its challenges and opportunities, ultimately improving outcomes for all stakeholders.

## Data Availability

Data used in the review is freely available.
